# Four studies exploring the growth and impact of heritage science as a discipline

**DOI:** 10.1038/s40494-026-02657-7

**Published:** 2026-05-28

**Authors:** Josep Grau-Bové, Ziwen Liu, Lana Nastja Anžur, Richard Higham, Emily Watt, Miriam Andrews, Matija Strlič

**Affiliations:** 1 University College London, Central House, 14 Upper Woburn Place, London, UK; 2 Heritage Science Laboratory, Faculty of Chemistry and Chemical Technology, University of Ljubljana, Večna pot 113, Ljubljana, Slovenia; 3 Natural History Museum, Cromwell Rd, South Kensington, London, UK; 4 McDonald Institute for Archaeological Research, University of Cambridge, Downing Street, Cambridge, UK

## Abstract

Heritage science has become a recognised discipline, and understanding its recent development is essential for shaping its future. This paper presents four complementary studies: a machine learning analysis of over 290,000 papers showing rapid expansion; a case study of 172 *Heritage Science* articles demonstrating interdisciplinarity; an examination of doctoral career trajectories; and a survey capturing professional perspectives on identity. Together, these studies provide a foundation for supporting the field’s continued growth.

## Introduction

Heritage science applies scientific solutions to heritage questions to ensure our future relationship with the past^1^. This type of research is by no means new, though only relatively recently has it been recognised as its own discipline (https://publications.parliament.uk/pa/ld200506/ldselect/ldsctech/256/256.pdf). Historically, heritage science research was segmented within conservation science, archaeometry, remote sensing, and material sciences^2^. In particular, archaeological science and heritage science were frequently conceptualised as separate domains, with differing institutional homes, research priorities and professional communities. However, by the turn of the millennium, it became increasingly apparent that there was a need to acknowledge and define heritage science as a standalone discipline capable of unifying these historically fragmented approaches under a shared interdisciplinary framework. In several national contexts, including the UK, this process of convergence has become increasingly pronounced in recent years. A growing awareness of the importance of heritage science research, together with recognition of the need to enhance resources, funding and skills, has led to greater clarity and consolidation of the field as a distinct entity.

Since this recognition, the discipline has expanded^2^, with considerable crossover with multiple disciplines such as geology, engineering, chemistry, biology and linguistic analysis^3^, as well as engagement with new digital technologies and techniques such as the use of artificial intelligence^4^. In the UK context, Heritage science is widely referenced in government advice and decision making in numerous different policy areas, ranging from government conservation strategies (https://assets.publishing.service.gov.uk/media/5a7f1e3140f0b62305b852e7/BAT_Heritage_Strategy_-_Nov_2016.pdf), repatriation of heritage (https://www.artscouncil.org.uk/supporting-arts-museums-and-libraries/supporting-collections-and-cultural-property/restitution-and-repatriation-practical-guide-museums-england), environmental guidelines (https://assets.publishing.service.gov.uk/media/5a7cc99740f0b6629523bcf8/7057.pdf), climate change adaptation policy (https://historicengland.org.uk/research/results/reports/6580/ClimateChangeAdaptationReport), as well as publications helping to unpack the public experience of heritage, cultural engagement, and sociological research (https://www.gov.uk/government/statistics/participation-survey-2023-24-annual-publication/main-report-for-the-participation-survey-may-2023-to-march-2024).

Present-day recognition and understanding of the importance of heritage science research is reflected in the investment into the formation of new infrastructures, established specifically to serve the field. These research infrastructures support the development of heritage science resources and innovation; for example, in the UK, the £22 million launch of the Research Infrastructure for Conservation and Heritage Science (RICHeS) (https://www.ukri.org/what-we-do/browse-our-areas-of-investment-and-support/research-infrastructure-for-conservation-and-heritage-science-riches/), indicates how government funding and recognition of Heritage Science has evolved since 2006. On an international level, the European Research Infrastructure for Heritage Science (E-RIHS) has recently been established as a European Research Infrastructure Consortium (ERIC). This ERIC is the result of the culmination of decades of international collaboration by heritage scientists, with the investment of 14 founding member countries. E-RIHS is based on an international recognition of the benefits of heritage science research and collaboration (https://www.e-rihs.eu/e-rihs-in-a-nutshell/).

Heritage science has evolved into a standalone discipline with substantial investment and recognition. It is beneficial, therefore, to understand how it has changed and if this affects the future outlook of the field, i.e. the key themes of scholarly outputs, areas for future engagement and research collaboration, as well as future training and technological requirements. This paper discusses the results of four separate but complimentary studies that chart the growth of heritage science research and its community. By presenting these studies in unison, it is possible to identify key themes about the discipline that help us imagine what the future of heritage science will look like. This, in-turn, helps determine how resources would be best used and on what training would be most useful for the next generation of heritage scientists.

Firstly, this paper presents a bibliometric study. This analysis assessed over 290 thousand publications on Web of Science (WoS), seeking to understand how the academic discourse on heritage science is expanding and changing. In recent years, many disciplines and areas of research have applied machine learning to open-source data to provide bibliometric insights into disciplines ^5–17^. Each of these studies follow a similar model: inputting large amounts of digitally available papers, usually from WoS, and using a machine learning language model to perform a word search over the key points of information. The names of institutions associated with researchers suggests the primary locations of research and the centres of different expertise, as well as indicating where collaboration is taking place. Keywords in titles and text also allow for key themes of research and gaps in knowledge to be identified. Machine learning language models are invaluable as they are able to process and screen large amounts of data for key points of information and allow a clear analysis of the field or research area. As such, the present study uses machine learning methods to quantify the present size of academic heritage science research and examine its growth.

This paper also analysed the disciplines that cite papers with a heritage science focus, using a case study of 172 *Heritage Science* journal articles published in 2020. From these two bibliometric studies, it is possible to ascertain:

- Which fields are impacted by Heritage Science research?
- What is the overall scientific impact in comparison to other fields?

The third study presented here analyses the careers and academic outputs of heritage science doctoral students who studied in the UK. A great need to train the next generation of heritage scientists has been identified due to the rapid expansion of heritage science as a discipline (https://www.heritagescienceforum.org.uk/documents/nhss_report_3_web.pdf). In the UK, this need led to the establishment of the SEAHA (Science and Engineering for Arts, Heritage and Archaeology) Centre for Doctoral Training (CDT) in 2014. This CDT was a collaboration between academic institutions, industry, and heritage partners, ensuring cross-disciplinary expertise that could shape the future leaders of heritage science research. SEAHA was funded by a £4.7million EPSRC grant and ran until 2023. In 2024, it is now possible to determine some of the impact of this funding by assessing the outputs of this ‘next generation’ of heritage scientists. Therefore, the third study analyses the outputs of the SEAHA graduate community, asking:

- Where do heritage science PhD graduates work?
- In what areas do they publish?
- What is their academic impact?

Lastly, an analysis is undertaken of a survey of heritage scientists that received over 413 responses between November 2022 and February 2023 (10.5281/zenodo.3946214) (https://www.iperionhs.eu/deliverables/D7.5-user-forum-and-organisation-report-on-activities). This survey, the Heritage Science Academy questionnaire, was undertaken as part of the project to establish the European Research Infrastructure in Heritage Science. It allows an in-depth exploration of the international heritage science community, its identity, the nature of the work and the places where research takes place, as well as engagement in training across an international setting.

The results of these studies are presented here and are used to identify emerging themes and future needs within this expanding interdisciplinary discipline. This aids reflection of achievements and may help to guide where future funding should be best invested.

## Bibliometric analysis

The bibliometric study uses heritage science publications to demonstrate how the field has evolved over time. Over 290,000 papers published between 2008 and 2022 were analysed using Natural Language Processing (NLP) techniques that classified academic articles as either related or unrelated to heritage or archaeological science. The methodology consists of four main steps: data preparation, text representation, dimensionality reduction, and classification.

### Data preparation and labelling

The initial dataset was sourced from the Web of Science using relevant keywords of 'heritage' and 'archaeology' for papers between 2008 and 2022. While the boundaries between heritage science and archaeological science are increasingly blurred in practice, their separate treatment here reflects historical patterns in publication practices and disciplinary labelling. This approach allows the degree of similarity in publishing trends between these two subfields to be examined, rather than assuming total convergence a priori. The details of ~143,000 papers related to heritage were scraped from the site, and 147,000 for archaeology. Naturally, this large sample includes many papers that use the word 'heritage' but are not related to heritage science.

To create a reliable training set, we manually labelled a subset of articles from 2008. A labelled dataset is needed (even for few-shot learning) because the performance of the model has to be assessed using ground truth labels. As such, the labelling strategy was as follows:

- Iterative labelling: we test the model after labelling some examples and decide if additional labelling is needed based on its performance.
- Focusing on 'hard' examples: we select data points that are more likely to confuse the model for labelling (for example, the term ‘heritage’ is also used in a lot of aeronautical papers).
- Keeping class balance: although this is a binary classification problem (whether the data belongs to the category or not), maintaining balance in each feature space is still essential.

Since the definition of heritage science is subjective and ambiguous, we sorted papers into both wide and strict categories. Papers that were considered to meet the strict definition of heritage science were those that had an interdisciplinary focus between heritage and STEM subjects, i.e. biology, chemistry, physics, engineering and computer science. The wide definition included all papers where a systematic methodology was used to study heritage. This included all the papers within the strict category, and also those where scientific approaches to heritage intersected with subjects like anthropology, sociology, geography, tourism studies and economics. We present only the results of the strict definition of heritage science here. Only one definition was used for archaeological science, which has a more specific remit: the application of scientific techniques and methods to the study of archaeological materials, sites and questions.

- First round of training: 100 papers were labelled as either heritage science with a wide and strict classification or marked as ‘not heritage science’; and 100 papers were labelled as archaeological science or marked as ‘not archaeological science’. From this, the first iterations of the models were generated.
- Second round of training: for both the heritage model and archaeology model, 30 additional papers were labelled as hard positives (topics that did not seem like heritage/archaeological science to the model but were considered to be upon expert evaluation); 30 additional papers were labelled as hard negatives (topics that seemed like heritage/archaeological science to the model but were considered not to be upon expert evaluation); and, 30 additional borderline papers (topics that the model classified as having a 50% probability of being heritage/archaeological science) were labelled as heritage or archaeological science or not. From this, the second iterations of the models were generated.
- Third round of training: the biases in the models were corrected by examining differences between the probability of papers being heritage science between iteration 1 of the model and iteration 2, and checking that any that had changed in their classification had been done so correctly. For this, we examined 300 additional papers for both the heritage science and archaeological science models, marking correct and incorrect assessments that were fed back into the models, generating the third iteration of the models.

A crucial step following the process outlined above was the inclusion of further obvious negative examples to expand and balance the training dataset. To identify obvious negative examples, we searched for articles that were obviously unrelated to heritage or archaeological science. This was done by examining specific keywords in their titles, source titles, or author keywords. We focused on terms from distinctly different fields, such as 'sport', 'medicine', 'nutrition', 'immunology', 'zoology', 'nursing', 'cancer', 'therapy' and 'patient', among others. To avoid potential false negatives, we carefully ensured that these negative examples did not contain any terms directly related to heritage or archaeology, such as 'heritage', 'historical' or 'cultural'.

The above approach allowed us to expand our training dataset with articles that were clearly outside the scope of our target fields, resulting in an additional 225 and 107 negative examples included in the model training sets for heritage and archaeology, respectively. After combining the manually labelled articles with the identified negative examples, our final training dataset consisted of 715 articles for the heritage science model and 597 articles for the archaeological science model. The fourth, and final, iteration of each model was thereafter generated.

### Text representation (Embedding)

To convert the text data into a format suitable for machine learning, we employed a technique called text embedding. This process transforms text into numerical vectors that capture semantic meaning. We used a pretrained model called ‘all-MiniLM-L6-v2’ [https://huggingface.co/sentence-transformers/all-MiniLM-L6-v2] to generate embeddings for each text field of the articles. This model, developed by the sentence-transformers project, is designed to create high-quality sentence embeddings.

We used five fields from each article for classification: Article Title, Source Title, Document Type, Author Keywords, and Abstract. These were considered the most relevant features, while others were not likely to be effective predictors for the classification of the paper. We used the embedding model to convert each field into a sentence embedding separately and concatenated them together as a unified representation of the article.

### Dimensionality reduction

The embeddings generated in the previous step are high-dimensional (384 dimensions per text field, amounting to 3845 = 1930 dimensions in total), which can be computationally expensive and prone to overfitting. To address this, we applied a dimensionality reduction technique called uniform manifold approximation and projection (UMAP). UMAP reduced the dimensionality of each text field’s embedding from 384 to 16 dimensions (165 = 80 dimensions in total) while preserving much of the data’s structure. This step made the data more manageable for the subsequent classification task.

### Classification model

For the final classification step, we employed an XGBoost [https://arxiv.org/abs/1603.02754] classifier, a powerful machine learning algorithm known for its performance in various classification tasks. The model was trained on the reduced-dimension embeddings of the labelled data.

To ensure robust evaluation with a limited size of our dataset, we used fivefold cross-validation. This technique involves dividing the data into five subsets, training the model on four subsets, and testing it on the remaining subset, repeating this process five times with different test subsets. This approach provides a more reliable estimate of the model’s performance on unseen data.

We evaluated the model’s performance using several metrics:

- F1 Score: A measure of the model’s accuracy that considers both precision and recall.
- Precision: The proportion of articles classified as relevant that are actually relevant.
- Recall: The proportion of actually relevant articles that the model correctly identified.
- Accuracy: The overall proportion of correct classifications (both relevant and irrelevant).

For the archaeology dataset, the model achieved an average F1 score of 0.7725, precision of 0.8039, recall of 0.7488, and accuracy of 0.8506. For the heritage dataset, the model achieved an average F1 score of 0.8062, precision of 0.8692, recall of 0.7603, and accuracy of 0.9298. For binary classification problems like ours (relevant vs. not relevant), the model outputs a probability between 0 and 1 for each article, representing the likelihood that the article is relevant to heritage or archaeology science. The probability threshold determines at what point we consider an article to be classified as relevant. In the final classification of articles from 2008 to 2022, we applied three different probability thresholds to categorise the articles:

- 0.5: A generous threshold, classifying more articles as relevant.
- 0.7: A moderate threshold, balancing between inclusivity and selectivity.
- 0.9: A strict threshold, classifying only the most confidently relevant articles.

This multi-threshold approach was adopted to address two key challenges in this classification task:

- the inherent subjectivity in determining whether an article is truly related to heritage or archaeology science, and
- the uncertainty inherent in any machine learning model’s predictions.

By providing results at different thresholds, we offer a more nuanced view of the field’s publication trends, allowing for flexibility in interpretation based on different levels of inclusivity.

### Results

**Fig. 1:**
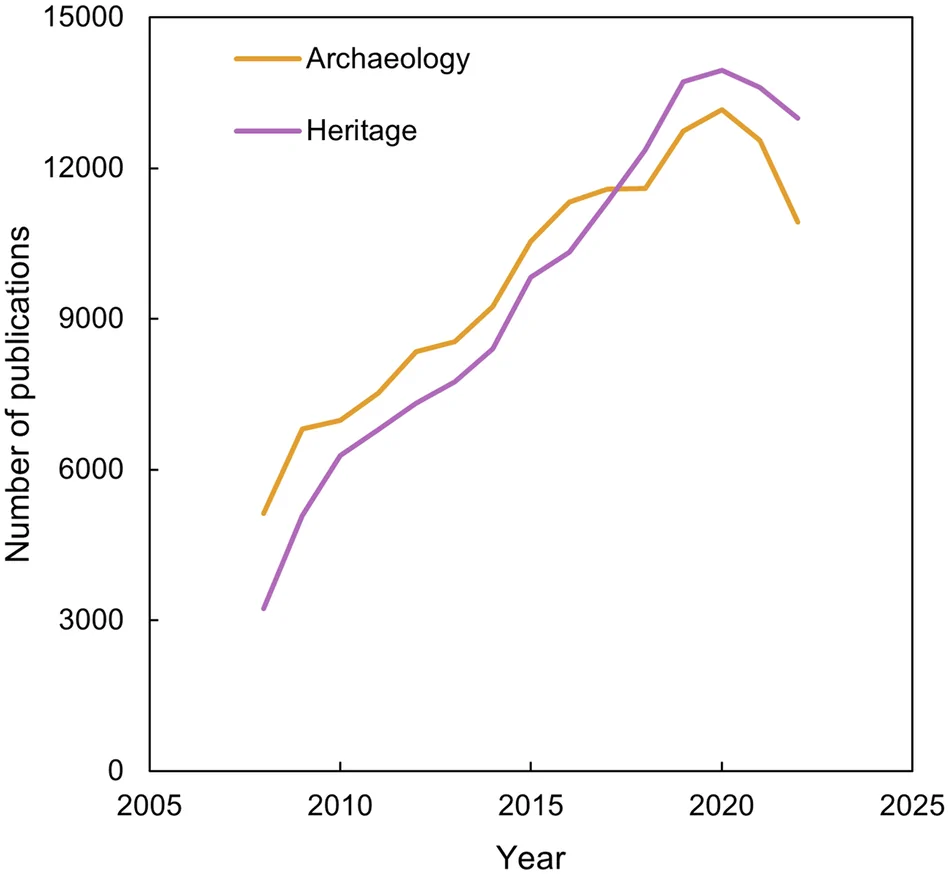
The number of publications containing the keywords ‘heritage’ or ‘archaeology’ from 2008 to 2022.

The analysis indicates that there is an increasing trend in the number of papers associated with the keywords ‘archaeology’ or ‘heritage’ within the sample of 290,000 papers scraped from Web of Science (Fig. 1). Given that the timeframe under analysis begins in 2008, this increase could be partially explained by the growing availability of digital publications.

The growth in publications including the keywords ‘archaeology’ or ‘heritage’ on WoS follows a similar trajectory, reflecting the comparable changes occurring across both fields and the likely overlap between them. Interestingly, from 2018 onwards, publications with ‘heritage’ as a keyword surpassed those in ‘archaeology’ as a keyword, reflecting the expansion of the use of heritage as a descriptor in scientific papers.

There was a decline in papers on WoS with the keywords of ‘heritage’ or ‘archaeology’ from 2020 to 2022. At first glance, this could be interpreted to correspond to the COVID-19 pandemic, which led to many academic endeavours being placed on hold or halted. Interestingly, a large 17-million-paper study covering 2019–2022 ^18^ found continued publication growth throughout the pandemic period, with no global collapse in output. However, output may have been unevenly distributed across topics and disciplines. A more persuasive explanation for the apparent shift in publication rates around 2020 is the modification of Clarivate’s article tracking methodology. Under the previous system, articles were attributed to the year of the journal issue in which they were formally assigned, even though many appeared online months earlier. For example, a paper published online in 2019 but allocated to a 2020 journal issue would have been recorded as a 2020 publication. When Clarivate implemented its revised tracking system, it produced an anomalously high publication count for one transitional year because online first articles were incorporated at the time of their initial appearance. This temporarily inflated publication volumes, and in some cases, impact factors, for that year, followed by an apparent decline in the subsequent year.

**Fig. 2:**
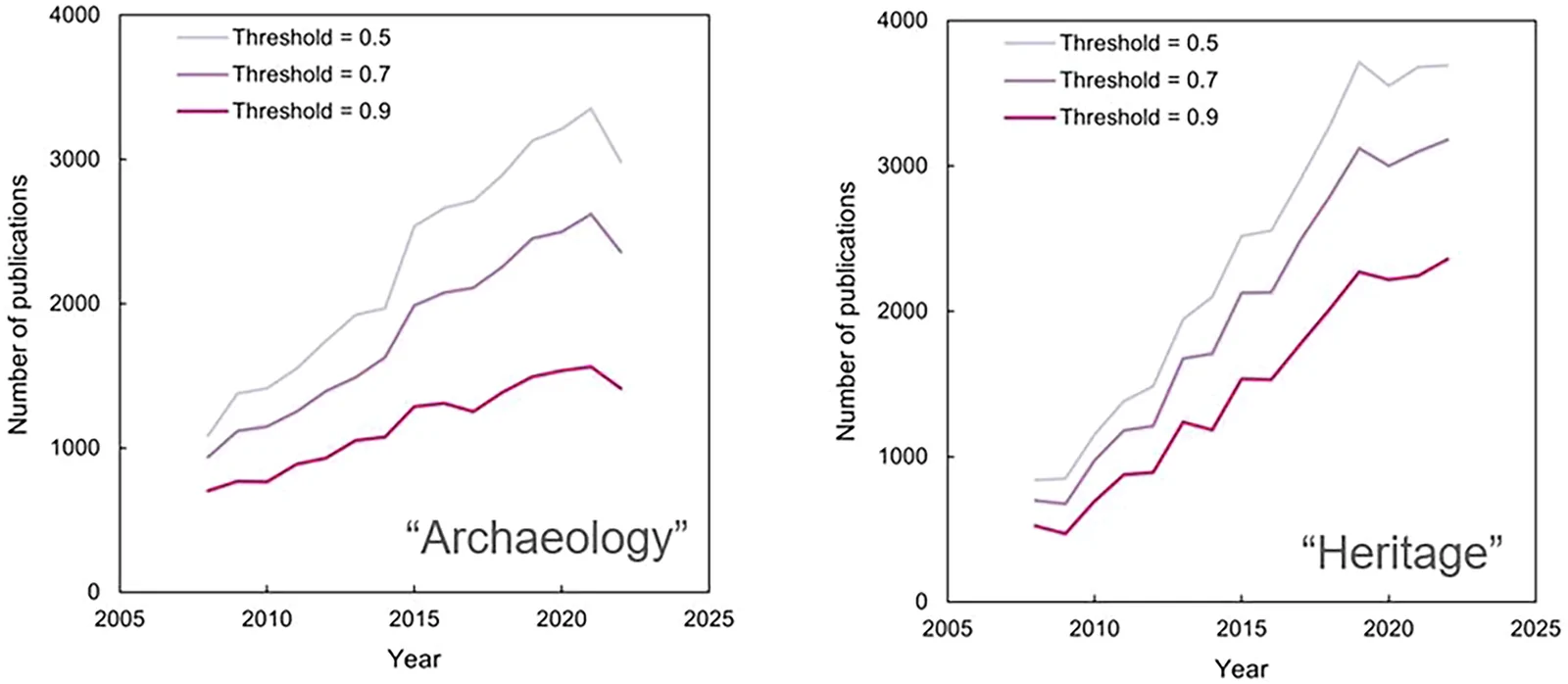
Model estimates of the number of archaeological science and heritage science papers based on different model thresholds.

The bibliometric model estimates fewer outputs than using keywords, suggesting that many of the papers listed on WoS are not actually founded in scientific methods (this was also noticed when manually labelling papers) (Fig. 2). When the generous 0.5 threshold is used, the models suggest approximately 3500 heritage science publications per year by 2022. At the 0.7 threshold, the output of the field is, 3000 papers per year by the end of the study period. At the 0.9 threshold, the output of the field is 2000 papers per year by the end of the study period. This likely represents a minimum value due to the various topics that were excluded by the strict definition.

## How are heritage science articles cited?

An in-depth analysis of how heritage science papers are cited allows for a greater understanding of the interdisciplinary nature and impact of the field. Using a case study of papers published in the journal of *Heritage Science* in 2020, it is possible to map their impact over the following 3 years.

The journal of *Heritage Science* published 172 articles in 2020. In the following 3 years, these articles were cited 704 times. Table 1 illustrates that 240 of these citations are within heritage science journals, while the rest are distributed across a wide range of subjects. In total, 463 (65%) of the citations are outside of the heritage science field (or 41% if art, history, and archaeology are counted as part of the heritage science discipline itself). Hence, this case study of papers demonstrates the interdisciplinary nature of the field and the relevance of heritage science topics to advancing knowledge and practice in other scientific domains.

**Table 1:** Research areas of journals citing heritage science publications

Journal research area	Number of citations
Heritage science	240
Chemistry	92
Engineering	89
Geography and environment	58
Imaging	57
General science	53
Archaeology	41
Biology	26
Physics	16
Computer science	10
Art	8
Medicine	5
Social sciences	4
Architecture	2
History	1
Mathematics	1

## Heritage science student study

A case study group of 63 SEAHA PhD heritage science doctoral alumni were contacted about their present field of study and work. The study was undertaken as a review of the success of the UK-based SEAHA PhD programme, which championed tackling social and scientific challenges across all scales of materials and environments, from climate change to molecular analysis. Not all SEAHA heritage science students were reached, and some failed to respond to the survey. This survey accounted for 29 alumni who responded, all of whom have found employment or continued further study in a wide variety of sectors where their heritage science skills are key to their roles. Some of the positions obtained by SEAHA alumni were directly related to heritage science, for example, Conservation Scientist at the Canadian Conservation Institute, Postdoctoral Researcher at the Getty Conservation Institute, and Digital Imaging Specialist at the Smithsonian. There were others that were outside the field, e.g. Journal Editor at Nature, Software Developer at Thoughtworks, Deputy Chief of Engineering at the Ministry of Justice and CEO of a company delivering services for retrofit. The diversity of the employment of students who have studied heritage science demonstrates the interdisciplinary nature of the subject and the relevance of the transferable skills gained within the SEAHA doctoral programme for roles within other sectors.

### Academic outputs

The students of the SEAHA Centre for Doctoral Training published 121 papers, which equates to 2.22 papers per student on average. This includes all the publications accumulated during the 10 years of the CDT (Sept 2014 - Dec 2023). These papers have been cited 1200 times.

## Heritage science questionnaire

The questionnaire was sent by email through a professional network of people working in fields related to cultural heritage science. Respondents replied between November 2022 and February 2023, and we received a total of 413 responses.

A total of 25.5% of respondents were aged 35–44, and 26.2% were between 45 and 54 years old. Close behind were those aged 25–34 (23.1%), typically considered young professionals. Participants aged 55–64 made up 18.3%, while the remaining respondents were distributed across the 18–24, under 18 and over 65 age groups. Most respondents were European residents (84%), followed by residents of South America (11%), North America (3%), Africa, Asia and Australia (all 1%). The majority of respondents (60%) held a PhD, followed by 27% with a master’s degree. Fewer participants reported holding a bachelor’s degree or professional qualification, each accounting for less than 6% of the total. The most represented type of occupation among all respondents was indicated as research science/technology/engineering/mathematics (35%), followed by conservators/restorers (20%), researchers in social sciences/humanities (13%), teachers and other educational roles (11%) and students (8%), with the remainder under 5%.

Almost half of the respondents (50%) work at least partly in the field of conservation, followed by chemistry (27%), archaeology and archaeological sciences (25%), art history (9%), physics (7%), art (6%), building sciences and engineering (6%). Other subjects account for less than 5% each. The most common combination is chemistry and conservation-restoration (10%), followed by archaeology and conservation-restoration (6%), archaeology & archaeological science or chemistry in 4%, art history or conservation-restoration in 3%, building science and engineering with conservation-restoration in 3%, physics in 2%, art and conservation-restoration in 2%, art and chemistry in 2%. Other combinations account for less than 1%.

### Heritage science identity

The survey indicates that the concept of heritage science plays an important role in the professional identities of the respondents. On average, 84% of respondents consider themselves to be heritage scientists. This is particularly relevant given the wide diversity of professional backgrounds represented in the sample. Respondents from the fields of archaeology, chemistry and conservation-restoration see themselves even more frequently as heritage scientists.

Those who are most likely to describe themselves as heritage scientists are at the PhD/early career stage. We can speculate on the reasons for the greater degree of self-declaration in younger age brackets; most likely, this is related to the development of specific heritage science degree programmes in the last decade. It seems that the establishment of an HS identity should be encouraged further in the 35–54 age group; these could be considered established professionals who are most likely to be in positions that allow them to make decisions and shape the policy, management, and strategic development of the heritage science sector. This non-registration might affect younger colleagues who are still at the beginning of their careers. One of the negative aspects expressed by younger members of the community is the lack of flexibility in adapting the nomenclature and systematisation of posts in the public sector. In academia, this may be reflected in the lack of appropriation of habilitation procedures for interdisciplinary fields such as sustainability, environmental studies and heritage science.

#### Identity and national organisations

There appears to be a meaningful relationship between the recognition of an HS identity and the presence of national HS organisations. A total of 73 membership-based associations and organisations active in the field were reported. Among respondents with memberships, 56% work in academic or research institutions, 24% in public or non-profit national bodies, and 11% in the private conservation sector. The remaining members are spread across various other types of workplaces. These findings suggest that future efforts should focus on encouraging greater participation from professionals outside academia in HS organisations.

### Students and young professionals (18–34)

Since one of our interests is to understand the needs of the field, which are crucial for successful solidification and development of heritage science, we focused the analysis on participants who can be considered students or early-career professionals. The majority reside in their country of origin, and work at academic institutions (73%). A significant proportion (20%) work in the public sector at the national level (non-profit). The rest work in public organisations at a local level (3%) or in museums and the private sector (both 1%). Most of them work in conservation (42%), though not at the exclusion of other disciplines. Among them are those who work only in conservation (37%) or in the combination of chemistry-conservation (31%) or conservation in combination with archaeology and archaeological sciences (9%), while other combinations (conservation/restoration in combination with other fields) account for 23%. Among other fields, we have respondents who work in chemistry-related fields (27%) or archaeology (21%). However, 10% of respondents aged 18–34 work in other fields, for instance, art, art history, anthropology, biology, climate science, computer science, law, materials science, medicine, physics, social sciences and economics.

### Reinforcing the heritage science sector and identity

Respondents were asked in a multiple-choice question which methods they would consider useful for strengthening the HS identity. In first place was funding, which was mentioned in 13% of responses. This was followed by conferences/networking events (11.74%), access to academic facilities/infrastructure (11.69%), education and training (10.04%), professional organisations (e.g. Society of Heritage Scientists) (9.22%), formal training in heritage science, e.g. BSc, MSc (8.57%), access to literature/learning resources and peer-reviewed Heritage science journals (8.10%) and public engagement (6.75%). The remainder were less than 5%. We should mention that the national-level public sector respondents have a slightly different preference. They have primarily indicated to be interested in access to both literature and infrastructure, followed by conferences.

**Fig. 3:**
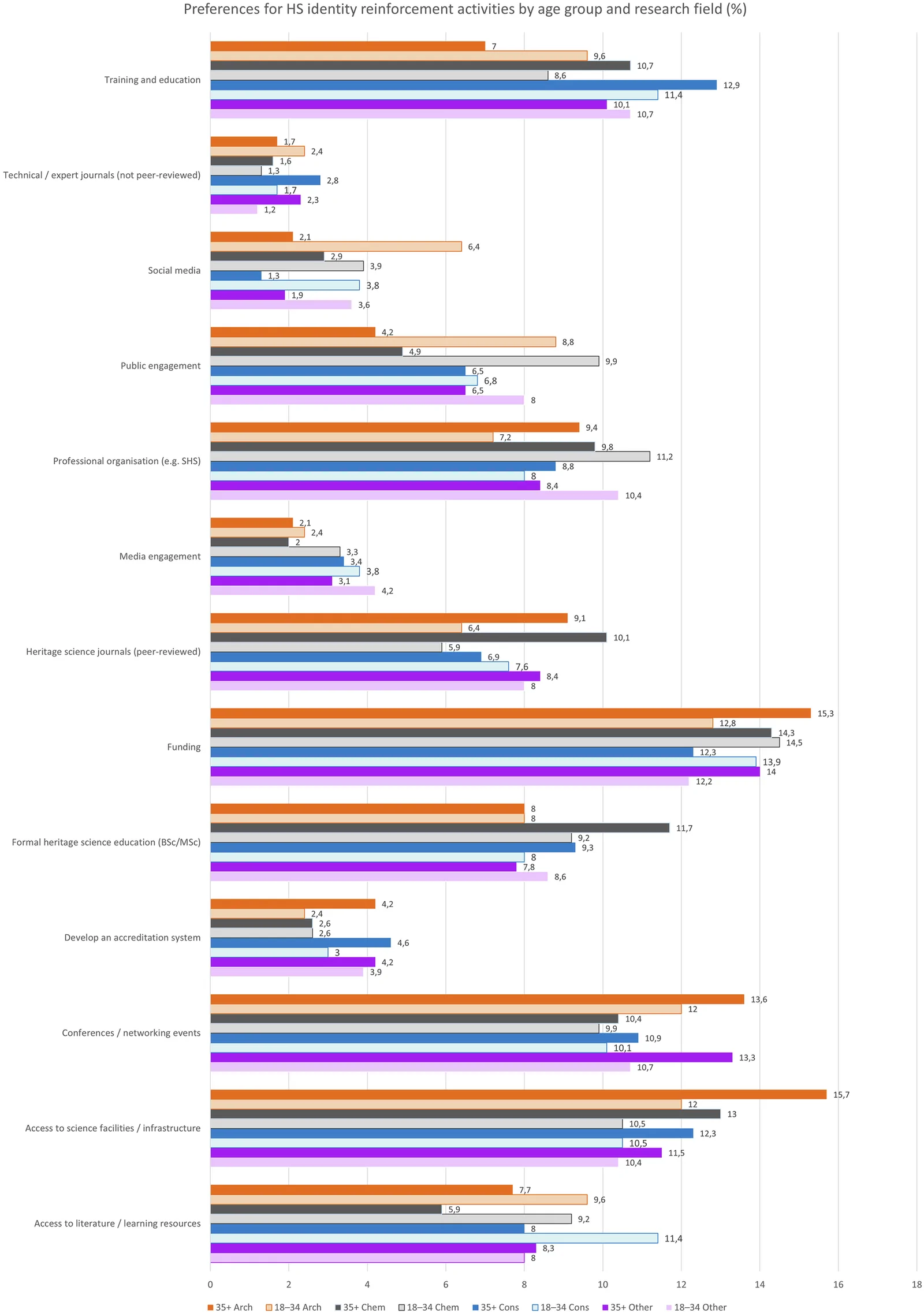
Preferences for Heritage Science identity reinforcement by age groups.

Further analysis was conducted to examine how the three most represented disciplines in the age group of established professionals (aged 35 years and above; 305 respondents or 73%) differ from each other and the general perspective on factors influencing identity formation within heritage science (Fig. 3). Among established professionals, 48% reported working in Conservation (Conservation-Restoration or equivalent), 24% in Chemistry, and 24% in Archaeology & Archaeological Science, with the remaining respondents representing other disciplines.

The largest discrepancy was found between the needs of respondents from the archaeological sector and others’ opinion on access to research facilities and infrastructure. This suggests they depend most strongly on physical infrastructure and that research infrastructure networks such as E-RIHS should be particularly encouraged among researchers in archaeology and the archaeological sciences. The next largest discrepancy was between the need for education and training for archaeologists and conservators. It seems that archaeologists feel well-trained but have difficulty accessing the necessary facilities, while conservators want to obtain more training in research. The general opinion, as well as that of the respondents from chemistry, lies between these two points.

It is also worth noting that the archaeological discipline was the only one where access to research facilities outweighed the need for funding. All other disciplines, as well as the overall opinion, indicated that funding was a higher priority than other needs, except in archaeology. A significant gap was found in the need for journals. Whilst respondents from the chemistry discipline felt that journals were needed, conservators felt that this need was better met. The results show that conservators place more importance, compared to chemists and archaeologists, on engagement in the media and define identity more strongly through skills development and training compared to archaeologists and chemists. A difference was also found in the need for formal education and peer-reviewed journals; chemists consider this to be more important than other fields.

We then divided the respondents into two groups: emerging professionals (18 to 34 years, 108 respondents), and established professionals (Fig. 3). Within the emerging professionals group, 53% reported working in Conservation, 33% in Chemistry, and 27% in Archaeology & Archaeological Science. Because respondents could select more than one field of work, percentages do not sum to 100% and reflect overlapping professional identities. The results clearly show that the younger population consistently places almost double the importance on the public, social media and media engagement. Young archaeologists are by far the most digitally networked and visible, while they are dependent on infrastructure. On the other side, chemists are most concerned with funding and link identity most strongly to public engagement. Young conservators define themselves most through training and skills. Results also show that in comparison to the younger population, established professionals place more weight on academic publishing and formal heritage-science education.

Findings suggest that younger people want the sector to have a stronger public presence overall. Another difference was younger people’s desire for good access to learning and training resources and funding. To summarise, we can say that in order to make the sector more attractive to the younger generation, we should focus on increasing public visibility and ensuring adequate funding, training and learning opportunities.

## Discussion

### Maturation and scientific impact of the field

**Fig. 4:**
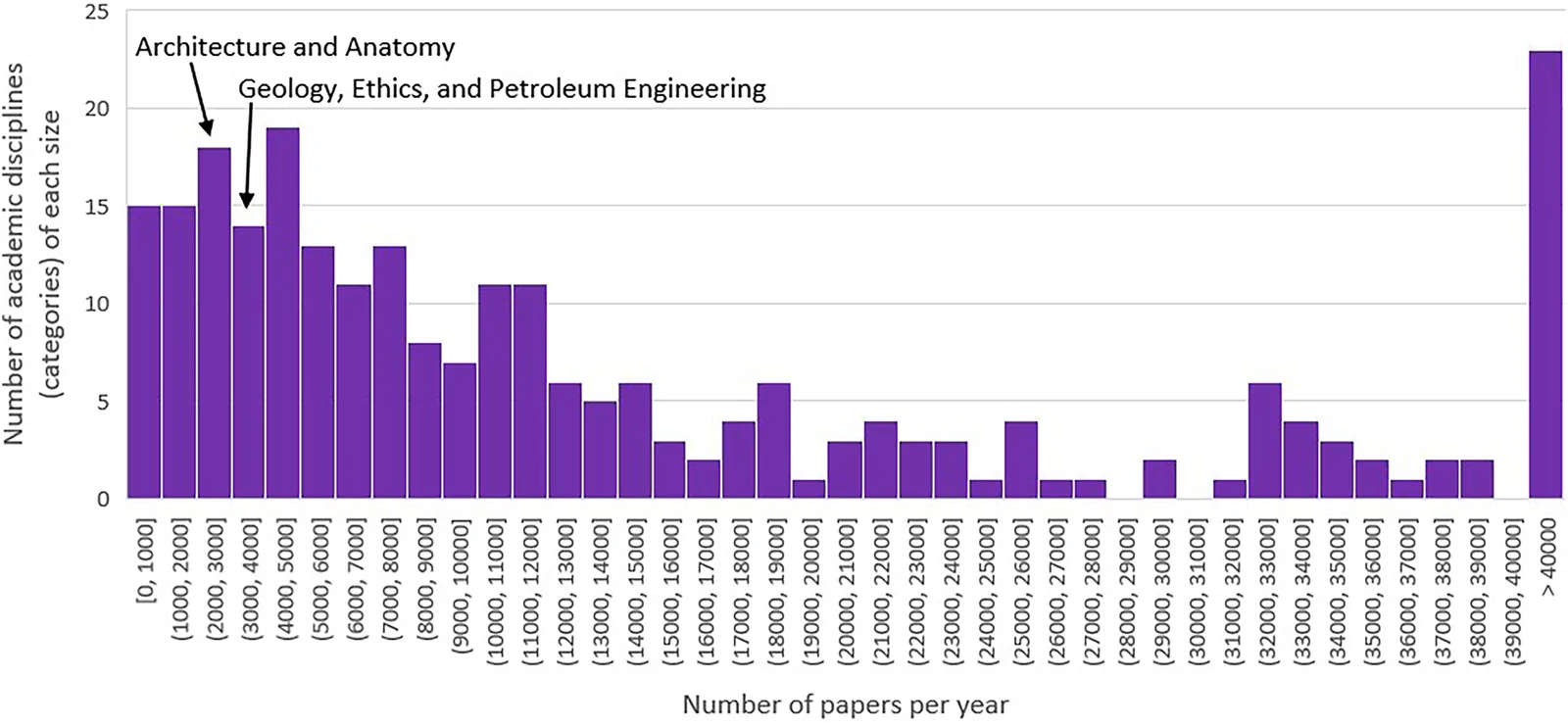
Number of papers published by different academic disciplines. Source used to create data within the Figure: Data from Web of Science, Journal Citation Reports (JCR), provided by Clarivate. Clarivate, WOS (Web of Science) and JCR (Journal Citation Reports) are trademarks and intellectual property of Clarivate and used herein with permission.

**Fig. 5:**
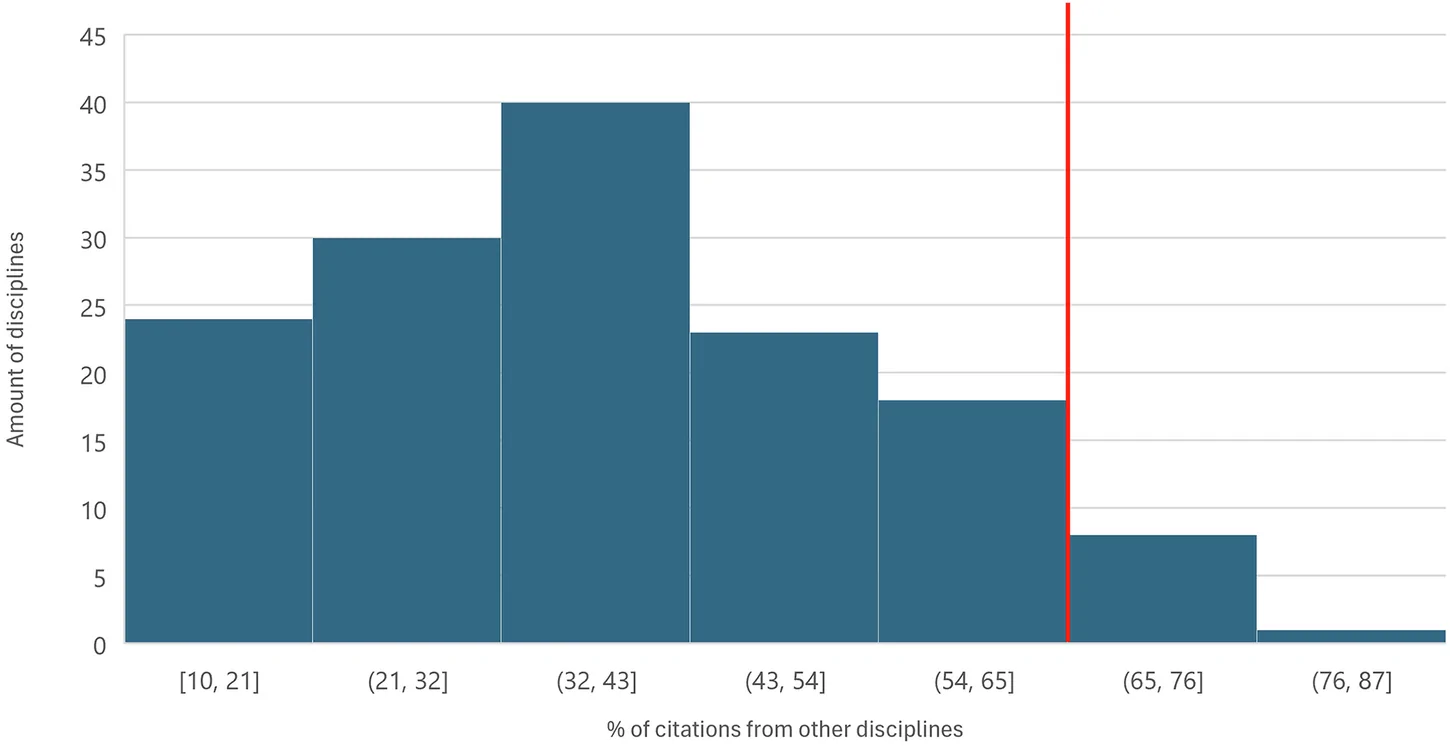
Plot demonstrating the interdisciplinarity of heritage science when compared to other subjects, with the red line showing the position of the field. Author-created figure using data from Van Noorden, R. (2015). Interdisciplinary research by the numbers. Nature, 525, 306–307. 10.1038/525306a. Data source cited; no original Nature figure elements reused^19^.

The output of the field has grown significantly over the last several decades, suggesting a substantial expansion of heritage science projects and activities. If we order all disciplines by the volume of papers generated each year, heritage science would rank at 50–60th (adopting the 0.5 or 0.7 model thresholds) out of 254 discipline/categories listed in Journal Citation Reports (Fig. 4). This puts the discipline on par with established fields like Geology, Ethics, and Petroleum Engineering, and above the fields of Architecture and Anatomy. As suggested by the case study of the papers published in the journal Heritage Science, the overall scientific impact of the field is wide, with almost two-thirds of citations originating from papers outside of the field. While long-established interdisciplinary partner fields were well-represented, e.g. chemistry and engineering, the papers were also cited frequently within environmental and imaging journals and even appeared within computer science and medical journals. As Fig. 5 suggests, this places heritage science in the top 26, if not the top nine, academic disciplines in terms of interdisciplinarity. This highlights the truly transferable nature of many scientific advances and innovations generated by heritage science research.

### Developing heritage scientists

Doctoral training programmes put in place to train the next generation of heritage scientists, for example, the SEAHA CDT in the UK, but also those funded under other collaborative schemes like the Una Europa alliance or by research councils, heritage organisations, and institutions have already made a significant contribution to the academic impact of the field. The results of the analyses conducted herein suggest that a structured programme coordinated by several interdisciplinary partners supports students in enhancing the scientific impact of their research.

The social return on the investment made in the formal training of heritage scientists in the UK is evident in the career prospects of completed PhD students, many of whom have secured further research positions or high-profile roles in the public, private, and third sectors. Some of these roles are within the heritage sector, while others are in other scientific domains, suggesting the importance of transferable skills gained within heritage science. As we will explore further below, one of the greatest strengths of the field is its inherent interdisciplinarity, which encourages students to appreciate wider perspectives, navigate complex interrelated systems, and draw upon diverse theoretical bases and methods to provide creative solutions. This inclusive, lateral-thinking approach produces ethically grounded, innovative scientists.

Largely as a result of the increasing infrastructure around heritage research, there has been a proliferation of informal training and continuing professional development opportunities available to heritage scientists. The HS Academy was launched by IPERION HS and E-RIHS partners in 2020, with the aim of addressing some of the gaps identified within the training provision for the E-RIHS community. The HS Academy offers several mediums and intensities of training to suit different types of professionals, users, and research communities with varying levels of involvement with heritage science, for example, webinars, doctoral training schools, and heritage science camps. However, the questionnaire reported here found that 54% of respondents were unaware of HS Academy, indicating that more work is required to raise the profile of these activities and ensure maximum impact. Other online resources are available from Parthenos, DARIAH, Una Europa, and eutopia, with topics such as interdisciplinary collaborative working, digital and data science research skills, research ethics, and compliance, particularly well-provided for.

### Forming the identity of the research community

Owing to the establishment of specific university courses for heritage science and investment in high-profile research infrastructures, there has been a consolidation of the identity of heritage science as a distinct field. The HS Academy questionnaire indicated that the majority of respondents identified themselves as heritage science scholars, with students and early-career professionals most likely to identify as such. However, it is worth noting that the questionnaire was promoted through E-RIHS channels and social media platforms. As such, the questionnaire primarily reached an audience already engaged with heritage science as a framing concept. It is therefore plausible that a similar questionnaire distributed primarily through more traditional archaeological science or conservation networks may have resulted in different patterns of disciplinary self-identification, reflecting ongoing variation in institutional structures and professional cultures. This raises the question of the extent to which the dispersion of the questionnaire reflects internal professional networks or the actual state of affairs. While responses were received from professionals in North and South America, Asia and Australia, the majority of respondents were based in Europe, likely reflecting the reach of the E-RIHS network. Whether, outside of Europe, heritage science is widely recognised as an overarching domain that unites subfields such as archaeometry, conservation science, and the analysis of the historic built environment, therefore, remains an open question.

As foreshadowed above, a key component of the heritage science identity is the diverse range of academic backgrounds that are brought together under a similar care and interest in cultural heritage and the historic environment. This blend of appreciation and understanding of the arts, humanities and social sciences, with scientific knowledge and technical skills, makes for extremely profitable collaboration and frequently allows heritage scientists to uncover what others may not perceive. Hence, it is no wonder that while the majority of the questionnaire respondents come from traditional heritage science adjacent fields, i.e. conservation, chemistry, and archaeology, there were many other tangential disciplines that were well-represented (physics, art history, building sciences and so on). Heritage science is the ideal field for researchers who find their curiosity pulling them across the various dimensions of academia; and, while specialism has its place, flexibility in thinking, which facilitates the cross-fertilisation of ideas, is also a highly respected attribute.

### Towards wider societal benefit

**Fig. 6:**
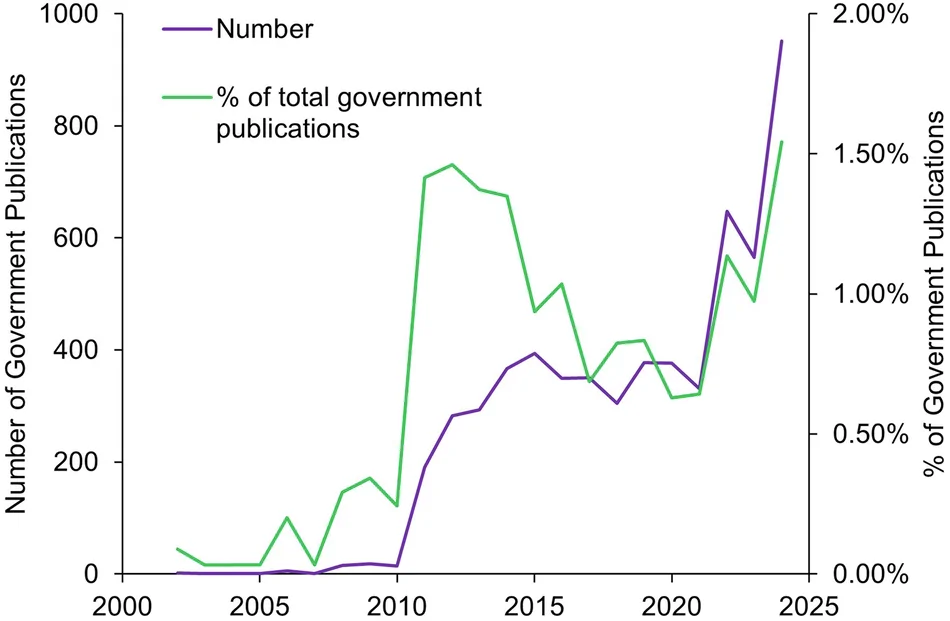
% of government publications in the UK that reference heritage. Reproduced from Kennedy, C.J.; Penman, M.; Watkinson, D.; Emmerson, N.; Thickett, D.; Bosché, F.; Forster, A.M.; Grau-Bové, J.; Cassar, M. (2024). Beyond Heritage Science: A Review. *Heritage*, 7(3), 1510–1538. 10.3390/heritage7030073, under the terms of the Creative Commons Attribution 4.0 International (CC BY 4.0) licence ^2^.

The translational ability of heritage science also increasingly extends to policy and social impact. For example, Fig. 6 illustrates the number of government publications in the UK that reference heritage, which has increased exponentially in the last several decades. In 2015, ICCROM determined that policy impact was an area of development for Heritage Science. Since then, the awareness of the potential impact of heritage science, especially in relation to climate change, the digitisation of heritage, wellbeing and cultural capital, has been growing within policy circles, with the UK providing a well-documented illustrative example. For instance, heritage science that makes use of climate modelling to understand the risks to cultural heritage has been pivotal in the production of the Fourth Climate Change Risk Assessment (led by the Met Office, on behalf of the UK Climate Change Committee) (https://www.ukclimaterisk.org/publications/technical-report-ccra4-ia/), which will be used to construct National Adaptation Plans for England, Wales, Scotland and Northern Ireland. In addition, the Department of Culture, Media, and Sport of the UK Government funded heritage scientists to work within their Culture and Heritage Capital Programme (https://assets.publishing.service.gov.uk/government/uploads/system/uploads/attachment_data/file/955203/GOV.UK_-_Framework_Accessible_v2.pdf).

### Future outlook

A key outcome of the survey was the identification of further training requirements, specifically on topics to meet arising academic and societal needs. When developing new degree programmes, it is important to emphasise the archaeological, chemical and conservation components in the curriculum, since it appears that the heritage science sector is most appealing to these profiles. There appear to be some knowledge gaps for individuals with a science education, who would benefit from additional knowledge in social sciences and computer science. It is crucial to incorporate subjects that address national conservation policy and integrate disciplines such as social sciences, economics, data science, computer science and climate science. This approach raises important questions about how personal development and effectiveness can be supported in relation to these interdisciplinary requirements.

As the field continues to grow in size and profile, the number of PhD students that will need to be trained to meet the demand of research activities will also increase. Some of these scientists will likely end up working in related fields, for which, they will need good transferable skills to ensure wide employability. In addition, perhaps it is time to rethink the existing nomenclature for positions to better reflect the diverse skill sets required in this evolving landscape. Cultural heritage became one of 28 ‘Blueprint for Sectoral Cooperation on Skills’ projects funded through the Erasmus+ programme. The project, named ‘Cultural Heritage Actions to Refine Training, Education and Roles’ (Charter), aims to address lifelong learning requirements in cultural heritage by (1) clarifying occupational roles and activities; and (2) the identification of curricula and learning outcomes to equip education and training to respond to current and future needs for cultural heritage skills (https://charter-alliance.eu/wp-content/uploads/2023/06/D3.4.-Report-Identifying-gaps-and-needs-in-the-educational-and-training-.rammes.pdf).

The survey also revealed key areas that could strengthen the sector. Overall, funding, infrastructure, and networking were considered the most essential for strengthening the identity of heritage science. When examining differences across disciplines, a notable gap was found between the archaeological sector and general opinion on the need for research facilities and infrastructure, suggesting that initiatives like E-RIHS should be particularly promoted within archaeology, while conservators identified a greater need for further training.

The data show that Heritage Science has matured into a dynamic and impactful field. This is evident across the main dimensions examined in this paper. The bibliometric analysis indicates a steady growth in the number of publications and a significant reach beyond the discipline itself. The study of a cohort of students reveals that heritage science training fosters employability both within and outside the sector, thanks to its interdisciplinary profile and transferable skills. A survey of the community further shows that a wide cross-section of disciplines now identify as part of heritage science.

However, this positive picture is not without challenges. In July 2025, the Declaration of the first conference of the National Heritage Science Forum in the UK voiced concern that, in a context of increasingly fractured geopolitics, heritage science should contribute through its ethos of understanding across disciplines. This is an ambitious goal, but as the findings suggest, it faces difficulties related to training gaps, particularly among younger generations. The current economic climate in many countries also hampers the creation of stable positions for heritage scientists, increasing the pressure to develop transferable and entrepreneurial skills.

The challenge for governments and funding bodies is to provide balanced support for the discipline, ensuring a complete training pipeline, from postgraduate education (including MSc and PhD programmes) to postdoctoral and technical positions, and ultimately leadership roles. Equally essential are sustained investments in physical and technical infrastructure, and in the translation of research into innovation, including entrepreneurship. All these strands of activity must be adequately supported. While the standing of the discipline continues to rise, the persistent challenge is that limited resources must be deployed strategically and with long-term vision.

## Ethical

The survey was conducted within the framework and ethical requirements of the IPERION HS project, which governed data protection. All participants were informed about the study aims and provided voluntary, informed consent prior to completing the survey.

## Data Availability

The datasets generated and/or analysed during the current study are not publicly available due to conditions that limit open sharing, but may be available from the corresponding author on reasonable request.

## Code availability

The underlying code for this study and training/validation datasets is not publicly available but may be made available to researchers on reasonable request from the corresponding author.
